# Screening for Ischemic Heart Disease with Cardiac CT: Current Recommendations

**DOI:** 10.6064/2012/812046

**Published:** 2012-09-18

**Authors:** Matthew J. Budoff

**Affiliations:** Los Angeles Biomedical Research Institute, Harbor-UCLA Medical Center, 1124 West Carson Street, Torrance, CA 90502, USA

## Abstract

Cardiovascular disease remains the leading cause of mortality in the US and worldwide, and no widespread screening for this number one killer has been implemented. Traditional risk factor assessment does not fully account for the coronary risk and underestimates the prediction of risk even in patients with established risk factors for atherosclerosis. Coronary artery calcium (CAC) represents calcified atherosclerosis in the coronary arteries. It has been shown to be the strongest predictor of adverse future cardiovascular events and provides incremental information to the traditional risk factors. CAC consistently outperforms traditional risk factors, including models such as Framingham risk to predict future CV events. It has been incorporated into both the European and American guidelines for risk assessment. CAC is the most robust test today to reclassify individuals based on traditional risk factor assessment and provides the opportunity to better strategize the treatments for these subjects (converting patients from intermediate to high or low risk). CAC progression has also been identified as a risk for future cardiovascular events, with markedly increased events occurring in those patients exhibiting increases in calcifications over time. The exact intervals for rescanning is still being evaluated.

## 1. Introduction

Imaging of atherosclerosis in the coronary arteries represents advanced atheroma and has been shown to be the strongest predictor of future cardiovascular (CV) events, outperforming traditional risk factors, inflammatory and other biomarkers, and other tests of atherosclerosis such as carotid intimal media thickness (CIMT), endothelial function, and ankle-brachial index. The traditional cardiovascular risk assessments underestimate the prediction of CV risk, and many individuals still suffer events in the absence of established risk factors for atherosclerosis [1]. Coronary artery calcium (CAC) has been shown to be the strongest independent predictor of future adverse cardiovascular events and also provides incremental information to the traditional cardiovascular risk factors assessment. It can be used to risk stratify asymptomatic individuals, improve the risk prediction provided by Framingham risk score (FRS), and follow the burden of calcified plaque over time, which is associated with further risk stratification beyond baseline score. 

## 2. CAC as a Risk Stratification Tool

There is tremendous evidence available that supports the role of CAC as the best risk stratifier for asymptomatic individuals [2]. CAC has been persistently shown to have superior independent and incremental predictor of CVD compared with traditional risk factors [3–13]. In the St. Francis Heart Study [3], a prospective population-based study of over 4000 persons followed for 4.3 years, a calcium score >100 predicted all atherosclerotic cardiovascular disease events, all coronary events, and the sum of nonfatal MI and coronary death events with relative risks of 9.5 to 10.7 at 4.3 years, as compared to patients with scores <100. This prospective study strongly demonstrated the ability to utilize this test to rule out patients who do not need therapy. CAC was predictive of coronary events, while highly sensitive CRP was not.

The Multi-Ethnic Study of Atherosclerosis (MESA), sponsored by the National Heart, Lung and Blood Institute, was a prospective population-based study of four different ethnic groups (whites, Hispanics, Asians, and African Americans) which provided a detailed insight into the role of CAC in risk assessment. Detrano et al. [4] showed that risk for coronary events increased by a factor of 7.73 among subjects with CAC scores between 101 and 300 and by a factor of 9.67 with CAC score above 300 when compared with CAC score 0 in 6,772 MESA participants. This 8-fold increase event rate was after multivariate adjustment for many factors, including C-reactive protein (which added no predictive value) and traditional risk factors. Across four ethnic groups, a doubling of CAC increased the risk of major coronary event by 15 to 35% and the risk of any coronary event by 18% to 39%. CAC provided an incremental value for prediction of major coronary events when added to the standard risk factors (0.83 versus 0.79 area under the curve for risk factors alone versus risk factors plus CAC, *P* = 0.006). Budoff et al. [5] showed CAC to be an independent predictor of mortality after controlling for age, gender, ethnicity, and cardiac risk factors (model chi-square = 2.017, *P* < 0.0001), in a registry of 25,253 asymptomatic individuals. CAC was shown to have significant incremental value compared with risk factors resulting in a higher concordance index (0.81 versus 0.61; *P* < 0.0001). Risk-adjusted relative risk ratios for CAC scores 11 to 100, 101 to 299, 300 to 399, 400 to 699, 700 to 999, and >1000 were 2.2-, 4.5-, 6.4-, 9.2-, 10.4-, and 12.5-fold when compared with CAC score 0, respectively (*P* < 0.0001). 

The South Bay Heart Watch [6] was the first study to compare the prognostic ability of CAC and highly-sensitive C-reactive protein (CRP). This study was a prospective observational population-based study on 1,461 asymptomatic adults with coronary risk factors who underwent CAC score and were followed prospectively for up to 8.5 years for nonfatal myocardial infarctions or coronary heart disease (CHD) deaths. This study demonstrated that CAC scores were incremental in predicting cardiac risk, and the relative risk (RR) of hard cardiac events (MI and death) increased from 1 to 4.9 with increasing calcium tertiles (*P* = 0.005), while highly sensitive CRP failed to add independent prognostic value for hard cardiac events. Compared with CAC score of zero, a CAC score of more than 300 was significant predictor (hazard ratio HR, 3.9; 95% confidence interval CI, 2.1–7.3; *P* < 0.001) of nonfatal myocardial infarctions or CHD deaths. Across FRS categories (0–9%, 10%–15%, 16%–20%, and ≥21%), CACS was predictive of risk among patients with an FRS higher than 10%  (*P* < 0.001) but not with an FRS less than 10%. FRS plus CAC resulted in a significantly greater mean area under the curve for the receiver operating characteristics curve compared with FRS alone (0.68 versus 0.63; *P* < 0.001). 

Kondos et al. reported a 37-month followup on 5,635 initially asymptomatic low- to intermediate-risk adults (mean age 51 ± 9 years) [7]. Patients with scores >170 had a relative risk for developing hard cardiac events of 7.24 (95% CI, 2.01–26.15), after multivariable analysis was performed with adjustment for age and other CAD risk factors. The presence of CAC provided incremental prognostic information over age and other risk factors.

A cohort of 10,377 asymptomatic individuals undergoing cardiac risk factor evaluation and CAC measure with EBT reported a mean followup of 5.0 years [8]. In a risk-adjusted model, CAC was an independent predictor of mortality (*P* < 0.001). This large observational data series shows that coronary calcium provides independent incremental information in addition to traditional risk factors in the prediction of all-cause mortality.

Taylor et al. studied a nonreferred cohort of healthy men and women aged 40 to 50 who were evaluated with measured coronary risk variables and coronary calcium detected with electron beam tomography [9]. Incident acute coronary syndromes and sudden cardiac death were ascertained via annual telephonic contacts, with a mean follow-up of 3 years. Coronary calcium was found in 22.4% of men and 7.9% of women. A total of 9 acute events occurred in men, including 7 of 364 men with coronary calcium (1.95%) and 2 of 1,263 men without coronary calcium (0.16%; *P* < 0.0001 by logrank). No events occurred in women. In these men, coronary calcium was associated with an 11.8-fold increased risk for incident CAD (*P* = 0.002) after controlling for the Framingham risk score. The study concluded that “In young, asymptomatic men, the presence of coronary artery calcification provides substantial, cost-effective, independent prognostic value in predicting incident CHD that is incremental to measured coronary risk factors.” Similar to multiple prior studies, addition of C-reactive protein added no clinical value.

More recently, the Heinz Nixdorf Recall (HNR) study performed a prospective, population-based study, and after 5.1 years, primary events occurred in 93 (2.3%) of 4137 participants (30% females) [13]. The event rate increased with the degree of CAC. Most importantly, the event rate in women with CAC ≥400 was similar to that in men (8.3% in 5 years). This means that the main determinant for CV risk is the degree of plaque burden. In the HNR study, the FRS reached an AUC of 0.681, and CAC improved the *c*-statistics significantly to 0.749 (*P* < 0.0003) [13]. 

A paper by Yeboah et al recently demonstrated that the best predictor of future CV events among any test or algorithm studied was CAC testing [14]. After 7.6-year median follow-up in MESA, CAC, ankle-brachial index, and family history were independently associated with incident CHD in multivariable analyses (HR, 2.60 [95% CI, 1.94–3.50]; HR, 0.79 [95% CI, 0.66–0.95]; HR, 2.18 [95% CI, 1.38–3.42], resp.). Carotid intima-media thickness and brachial flow-mediated dilation were not associated with incident CHD in multivariable analyses (HR, 1.17 [95% CI, 0.95–1.45] and HR, 0.95 [95% CI, 0.78–1.14]). Coronary artery calcium afforded the highest increment in improvement in AUC (0.623 versus 0.784, *P* < 0.0001). They concluded that CAC provided superior discrimination and risk reclassification compared with other risk markers. 

CAC has been shown to have superior predictive and incremental value in risk stratification of asymptomatic individuals. It has been suggested for use in risk stratification of intermediate risk, low to intermediate risk, and diabetic individuals in recent AHA/ACC guidelines [2, 15]. The role of baseline CAC score may provide a useful option to strategize preventive therapeutic and aggressive treatment strategies due to its ability to reclassify low-risk populations as determined by the traditional risk assessment tools. 

## 3. Net Reclassification with Coronary Artery Calcification

Pencina et al. [16] introduced the concept of net reclassification improvement (NRI) which measures the extent to which people with and without events are appropriately reclassified into clinically accepted higher- or lower-risk categories with the addition of a new marker. CAC has been shown to reclassify the risk assessment based on FRS and National Cholesterol Education Adult Treatment Panel (ATP) III guidelines into either higher- or lower-risk categories based on CAC score information [13, 17, 18]. The net reclassification improvement in MESA for the entire cohort was 25% [15]. Those who were upgraded had in 16.4% an event, and those who were downgraded had an event in 2.3%. In the intermediate risk score cohort, 16% were reclassified at high risk and 39% at low risk which resulted in a net reclassification improvement of 55% [17].

A more recent analysis from MESA with longer follow-up [14] demonstrated net reclassification of almost 66%. For incident CHD, the net reclassification improvement with CAC was 0.659, orders of magnitude were better than brachial flow-mediated dilation 0.024, ankle-brachial index was 0.036, carotid intima-media thickness was 0.102, family history was 0.160, and high-sensitivity CRP was 0.079. In the HNR study, 57% of male and 64% of female subjects in the intermediate group were reclassified as the low-risk category [13]. Only 6.6% of the total intermediate group remained in the intermediate category and would need reassessment during follow-up or additional analysis of signs of atherosclerosis in other vascular territories. The HNR study yielded a net reclassification improvement for hard coronary events of 30.6% for the 6%–20%/10-year risk threshold. There was significant improvement in the area under the curve from 0.681 to 0.749 (*P* < 0.003) and from 0.653 to 0.755 (*P* = 0.0001) by adding CAC scores to the FRS and National Cholesterol Education Panel ATP III categories, respectively. Also the Rotterdam study presented the effect of reclassification in the intermediate risk group defined as 10%–20% 10-year risk [18]. A total of 52% of men and women were reclassified, all into more accurate categories. In asymptomatic patients, the S. Francis Heart Study demonstrated reclassification in 73% of intermediate-risk patients [3]. 

In the MESA study, 23% of those who experienced events were reclassified to high risk, and 13% without events were reclassified to low risk using CAC in addition to traditional risk factors [17]. Also, 90% of women in MESA study were found to be at low risk. In this group, >30% of women were found to have CAC score >0, whereas the prevalence of significant CAC (≥300) was four percent [12]. These subjects would have not received preventive therapeutic treatments based on traditional risk factor assessment alone. However, the use of CAC in these populations may be helpful to identify individuals who are in need of these therapeutic treatments. Serial measurement of CAC over time also provides useful information about the coronary risk of these individuals. Multiple guidelines support the utility of CAC in asymptomatic persons to risk stratify individuals. Use of CAC in symptomatic populations has been demonstrated to provide a cost-effective filter prior to invasive angiography [15, 19]. 

## 4. Absence of Coronary Artery Calcium

The risk for future adverse cardiovascular events increases with increasing CAC scores; however, the absence of CAC presents a very unique situation which is associated with very low-risk status for the individual (10-year event rate of ~1%) [2, 11, 15, 20]. It has been proposed that those without calcification may be at such a low-risk status, that further intervention with pharmacology may be unnecessary. The absence of CAC is associated with a very low risk of future cardiovascular events, presence of severe CAD, myocardial perfusion abnormalities and likelihood of ACS. The absence of CAC on noncontrast CT identifies an extremely low-risk population and potentially can serve as excellent indicator of low likelihood of present and future risk of CAD. For symptomatic persons, the presence of CAC was highly sensitive (98%) in predicting a luminal stenosis >50% in any coronary artery. In fact, recent ACC/AHA guidelines also consider that “for the symptomatic patient, exclusion of measurable coronary calcium may be an effective filter before undertaking invasive diagnostic procedures or hospital admission” [15, 19].

Furthermore, data in patients undergoing myocardial perfusion scanning (MPS) also have low risk when CAC scans show no calcifications. One prognostic study simultaneously evaluating the prognostic value of both MPS and CAC scores conclusively showed that the event risk of a person without CAC was extremely low regardless of whether they had ischemia or not [21]. In fact, none of the patients without CAC who had an abnormal MPS suffered an event, while a very low percentage of those without CAC and a normal MPS suffered an adverse outcome (0% versus 0.2%, resp.). The 2010 ACCF appropriateness criteria state that a low calcium score (especially in the absence of CAC) precludes the need for MPS assessment [22], and this is reiterated in the 2010 ACCF guidelines [2].

For event-driven analysis, Blaha et al. [11] showed that there were 104 deaths (0.52% event rate) among patients with CAC score zero (19,898 patients) compared with patients with CAC score >10 (3.96% event rate). The annualized all-cause mortality rates for CAC score zero and CAC >10 were 0.87 and 7.48 deaths/1,000 person-years. CAC score of zero separates the very low-risk group from subjects that are at relatively higher risk for future adverse cardiovascular events whether the amount of CAC is minimal or significant. Budoff et al. [23] performed an analysis in a multiethnic population of MESA study where they showed that the CAC score of zero (3,415 individuals) was associated with 17 CHD events (0.1%/year) compared with 11 in those with CAC of 1 to 10 (2% event rate). In age- and gender-adjusted analysis, presence of minimal CAC (1 to 10) was associated with an estimated 3-fold higher risk of a hard CHD event (HR: 3.23; 95% CI: 1.17–8.95) which remained robust even after adjustment for traditional cardiovascular risk factors and carotid intima media thickness. Raggi et al. [24] followed 632 patients for a mean of 32 ± 7 months for the development of acute myocardial infarction or cardiac death. A zero score was associated with a 0.11% per year event rate, compared to 4.8% per year with a score >400. They demonstrated the incremental benefit of adding calcium scores to conventional risk factors. Multiple logistic regression analyses demonstrated that the CAC score percentile was the only significant predictor of events and provided incremental prognostic value when added to traditional risk factors for CAD (chi-square, *P* < 0.001). This study both demonstrated the prognostic importance of a high score and the potential to rule out CAD with a CAC of zero. Greenland et al. [2], in a summary of multiple studies, showed that asymptomatic patients with CAC score zero had a low event rate (0.1%/year) over the 3 to 5 years period in ACC/AHA clinical consensus document. Similarly, in MESA study, subjects with CAC score 0 had an exceptionally low CVD event rate in an ethnically diverse subject population [4]. Due to extremely low event rate in subjects with CAC score of zero, it is being used to exclude CAD as the cause of chest pain in low risk patients in NICE clinical guidelines in Britain [25].

## 5. CAC in Lower-Risk Populations

Traditional risk factor assessment has been shown to underestimate coronary risk in certain population groups especially women and young adults [26–28]. This may lead to suboptimal medical management for their coronary risk factors. CAC may help to refine the risk stratification and treatment strategies of these patient populations who are otherwise categorized as low risk based on FRS categorization. Among 3,601 women in MESA study [12], 90% of women were in the low-risk category according to FRS categorization after excluding women with diabetes and older than 79 years of age. In this group, >30% of women were found to have CAC score >0, whereas the prevalence of significant CAC (≥300) was four percent. The hazard ratio of coronary heart disease in women with CAC score >0 was 6.5 (95% CI; 2.6–16.4) compared with women with CAC score of zero. Berry et al. studied incidence and progression of subclinical atherosclerosis in young adults ≤50 years of age in subjects enrolled in Coronary Artery Risk Development in Young Adults (CARDIA) and MESA study [29]. They found that individuals with low 10-year but high lifetime risk have a greater subclinical atherosclerotic burden on carotid IMT and CAC and greater incidence of atherosclerotic progression compared to individuals with low 10-year and low lifetime risk. In light of these and other data, current practice guidelines suggest that physicians consider current risk factor burden within the context of long-term or lifetime risk for CVD [30–32]. 

## 6. Risk Benefit of Coronary Artery Calcification

CAC is a noninvasive measure of atherosclerosis; however, its use is associated with certain radiation exposure and medical cost. Budoff et al. [33] evaluated cost of CAC testing compared to MPS algorithms for evaluation of CAD after borderline or abnormal exercise treadmill test. Evaluating patients with abnormal treadmills led to direct cost savings of $873 per patient for the baseline case. They concluded that CAC followed by selective CTA-based strategies lowered overall health care costs, primarily by decreasing the rate of false-positive examinations, thereby leading to fewer angiograms. Rozanski et al. [34] performed a prospective, randomized trial evaluating the direct impact of CAC on future CAD risk and downstream medical cost compared to that of the conventional medical practice, in the EISNER (Early Identification of Subclinical Atherosclerosis by Noninvasive Imaging Research) study. They evaluated 2,137 volunteers to undergo CAC scanning or randomized not to undergo CAC scanning before risk factor counseling. The primary end point of the trial was 4-year change in CAD risk factors and FRS. Compared with no scan group, the scan group showed a net favorable change in systolic blood pressure (*P* = 0.02), low-density lipoprotein cholesterol (*P* = 0.04), and waist circumference for those with increased abdominal girth (*P* = 0.01) and tendency to weight loss among overweight subjects (*P* = 0.07). FRS remained static in the scan group compared with no scan group (0.002 ± 4.9 versus 0.7  ±  5.1, *P* = 0.003). Within the scan group, increasing baseline CAC score was associated with a dose-response improvement in systolic and diastolic blood pressure (*P* < 0.001), total cholesterol (*P* < 0.001), low-density lipoprotein cholesterol (*P* < 0.001), triglycerides (*P* < 0.001), weight (*P* < 0.001), and FRS (*P* = 0.003). The EISNER study also prospectively evaluated the procedural cost and resource consumption patterns after CAC measurement during the 4-year followup period using Medicare reimbursement rates. They showed that downstream frequency of medical tests and costs was significantly lower in those persons who had CAC zero as compared to those who did not receive a CAC scan. 

LaMonte et al. [35] studied 10,746 adults aged 22 to 96 years and free of known CAD. The individuals were studied by electron beam tomography (EBT) for coronary calcium assessment as part of a preventive medical examination. During a mean follow-up of 3.5 years, 81 hard events (CAD death, nonfatal MI) and 287 total events (hard events plus revascularization) occurred. The age-adjusted rates of hard coronary events per 1,000 person-years in four categories of calcium scores (0, 1–99, 100–399, 400, or above) were 0.4, 1.5, 4.8, and 8.7 for men and 0.7, 2.3, 3.1, and 6.3 for women (significant in men and women). Even after risk factor adjustment, the association between calcium and events remained significant, and significant cost savings were appreciated.

## 7. CAC Progression

While baseline scores have proven clinically and prognostically important, cost effective, and better than other markers of CV risk in all studies published, data on progression of CAC is more divergent. The most comprehensive study of factors associated with progression of CAC was the MESA study [36]. In the MESA cohort, many traditional cardiovascular risk factors were associated with both the risk of developing incident coronary calcium and increases in existing calcification. These included older age, male gender, white race/ethnicity, hypertension, higher body mass index, diabetes mellitus, and family history of heart attack. Diabetes mellitus was the strongest risk factor for CAC progression. In fact, glucose was associated with higher risk of incident CAC even at levels well below the standard thresholds for diabetes mellitus or impaired fasting glucose and among nondiabetic participants. Among treated diabetics, duration of diabetes was the only significant risk factor for CAC progression after adjustment for age, gender, scanner, and duration of follow-up. Certain factors also appeared to be related only to the risk of incident CAC but not to the progression of existing calcification. For instance, LDL cholesterol and HDL cholesterol were not associated with the progression of existing CAC, but both were associated (HDL negatively) with the risk of incident CAC. Triglycerides were associated with both end points in models adjusted for age, gender, and follow-up time, but in fully adjusted models, this association became nonsignificant for CAC progression while remaining significant for incident CAC. Although both fibrinogen and CRP were related to an increased risk of incident CAC, this became nonsignificant after adjustment for body mass index.

### 7.1. Predictive Value of Serial CAC Score Progression

The real question becomes as follows: “are CAC progressors at increased risk for CVD, or is CAC progression just a representation of increased scar tissue?” Atherosclerosis is a dynamic process which may be affected by traditional risk factors, environmental factors, and therapeutic interventions [36–39]. Baseline CAC represents a single point in time on atherosclerosis time curve, whereas progression presents the slope of that curve. CAC progression may provide insight into ongoing atherosclerotic disease activity. Therefore, CAC progression might represent a better marker of disease activity a better predictor of future adverse cardiovascular events and can be used to evaluate the effect of various therapeutic interventions. Measurement of CAC progression highly depends on accurate reproducibility of CAC scores, which will be discussed in detail in a later section [37, 38]. 

Budoff et al. showed that CAC progression was significantly associated with mortality even after adjusting for baseline CAC, age, sex, time between scans, hypertension, hypercholesterolemia, diabetes, family history, and smoking (hazard ratio = 3.32, 95% CI: 2.62–4.20, *P* < 0.0001) in 4,609 consecutive asymptomatic individuals undergoing two scans. Adjusted models showed that the presence of CAC at baseline and associated significant progression of CAC was a significant predictor of future mortality (HR: 5.33, 95% CI: 3.74–7.60, *P* < 0.0001) [40]. This study also evaluated various methods of CAC progression estimation and their performance in predicting future mortality (absolute score difference, percent annualized difference between baseline and followup scans, and the difference between square root of baseline and square root of followup CAC score >2.5 (the SQRT method)). The SQRT method was found superior followed by percent increase in CAC score (>15% yearly increase) in predicting mortality [41]. Raggi et al. [42] in an observational study related the occurrence of acute myocardial infarction to CAC progression in 817 asymptomatic individuals referred for sequential CAC scanning (average interval 2.2 ± 1.3 years). Mean absolute and percent changes in CAC score were 147% and 47%, respectively, in those who developed a myocardial infarction (MI) after the second scan, compared to 63% and 26% in those without events (*P* < 0.001 and *P* = 0.01, resp.). In this study, CAC progression was the strongest predictor of myocardial infarction. In another study with 495 asymptomatic subjects undergoing sequential CAC scans and statin therapy after the initial scan, Raggi et al. showed that subjects having myocardial infarction had a greater annual change of CAC scores compared with those who did not had an event (42%  ±  23% versus 17%  ±  25%, *P* = 0.0001), respectively [43]. Relative risk of having myocardial infarction in the presence of CAC progression was 17.2-fold (95% CI: 4.1 to 71.2) higher than without CAC progression (*P* < 0.0001). The follow-up CAC score (*P* = 0.034) and a score change >15% per year (*P* < 0.001) were shown to be independent predictors of time to MI. The St. Francis Heart Study [3] prospectively evaluated the prognostic accuracy of CAC progression in the prediction of cardiac events in 4,613 adults between 50 and 70 years of age. Follow-up was over 4.3 years, and events occurred in 119 subjects (2.6%). Median increase in CAC score was significantly higher in those with cardiac events compared with those without events (247 versus 4), respectively. In multiple logistic regression, two-year change in calcium score (*P* < 0.0001) was the strongest association with subsequent CAD events. Several smaller studies have also been reported. Raggi et al. followed 269 asymptomatic subjects for 2.5 years after being submitted to sequential CT scans; of the 22 CVD events, 20 occurred in patients with progression of CAC and 2 without progression (*P* < 0.01) [44]. Another study of 225 subjects followed for an average of 3 years after the repeat CT scan, and those with new cardiac events had a significantly greater annual change in CAC score than those who did not experience events (35% versus 22%, *P* = 0.04) [45]. Moreover, 78% of patients with events had >20% annual progression versus 37% of those not experiencing events (*P* < 0.001). 

The largest and most comprehensive study of progression has just been reported. The Multi-Ethnic Study of Atherosclerosis (MESA) has just reported the outcomes associated with progression of CAC [46]. MESA studied 6,778 persons (52.8% female) aged 45–84 years from the Multi-Ethnic Study of Atherosclerosis. 5,682 persons had baseline and follow-up CAC scans approximately 2.5 ± 0.8 years apart; multiple imputation was used to account for the remainder (*n* = 1,096) missing follow-up scans. Median follow-up duration from the baseline was 7.6 (max = 9.0) years. CAC change was assessed by absolute change between baseline and follow-up CAC. Cox proportional hazards regression providing hazard ratios (HRs) examined the relation of change in CAC with CHD events, adjusting for age, gender, ethnicity, baseline calcium score, and other risk factors. 343 total and 206 hard CHD events occurred. The annual change in CAC averaged 24.9 ± 65.3 units. Among persons without CAC at baseline, (*n* = 3,396), a 5-unit annual change in CAC was associated with an adjusted HR of 1.4 (1.0–1.9) for total and 1.5 (1.1–2.1) for hard CHD. Among those with CAC >0 at baseline, HRs (per 100-unit annual change) were 1.2 (1.1–1.4) and 1.3 (1.1–1.5), respectively. Among participants with baseline CAC, those with annual progression of ≥300 units had adjusted HR's of 3.8 (1.5–9.6) for total and 6.3 (1.9–21.5) for hard CHD compared to those without progression. They concluded that progression of CAC is associated with an increased risk for future hard and total CHD events.

These studies suggested that continued accumulation of CAC in asymptomatic individuals is associated with increased risk of future MI. The strength of serial CAC assessment is to evaluate the ongoing activity of atherosclerosis, while CAC offers a measure of lifetime accumulation of atherosclerosis. However, measure of progression is dependent upon a positive (nonzero) calcium score. Baseline zero scores were not predictive of progression or all-cause mortality in the MESA study [46]. This further validates the concept that a baseline zero score has a significant warranty period for both future cardiovascular events and progression of atherosclerosis. Studies have suggested that a zero calcium score affords at least a 5-year warranty period [45–48]. In the study by Min et al. [48], the cumulative rate of “conversion” from a zero CAC score to ≥1 CAC score was 15% in the first 4 years and 25% in the fifth year concluding that 4 years might be the ideal “warranty period” for a zero CAC score which is considerably longer than the warranty period that is offered by normal functional imaging tests (such as nuclear perfusion scans) which is considered to be around 1.5 to 2 years [49]. A 5-year follow-up study by Gopal et al. [47] showed that only 2% of patients with baseline zero calcium score had CAC progression >50 suggesting that in individuals with no detectable CAC on an initial scan, a repeat scan can be recommended no sooner than 5 years. 

### 7.2. Pathogenesis of Progression

The presence of calcium in coronary arteries is pathognomonic of atherosclerosis, but the pathogenesis of increasing CAC is still under some debate [50, 51]. The close correlation between the atherosclerotic plaque burden and the extent of CAC has been confirmed by both histopathology and intravascular ultrasound [52–55]. While CAC detected and quantified by CT represents an accurate anatomic measure of plaque burden, it only represents approximately 20% of total coronary plaque burden [56, 57]. Assessment of CAC progression has been regarded as a dynamic measurement that might provide insight into ongoing current disease activity and more efficiently predicts future cardiac events, by its association with increased total plaque burden, rather than static traditional clinical parameters and baseline CACS [58, 59]. CAC has been widely suggested as a tool to monitor the progression of coronary plaque burden and to document the success of risk factor modification and medical intervention [60, 61]. 

### 7.3. Calcium Score Reproducibility

 Typically, coronary calcium, untreated, progresses at 10% of the baseline value per year, although data suggest that a progression rate of >15% per year is associated with a 17-fold increased risk for incident CAD events [42]. The accuracy and reproducibility of the test are paramount to use serially to monitor effects of therapy [37, 38]. There have been multiple studies of reproducibility done using CAC, starting with the earliest iterations of electron beam tomography and progressing to 64+ multi-detector computed tomography. Early studies have demonstrated mean reproducibilities of 14–38% using scanner or scanning algorithms that are no longer up to date [61–67]. Improvements in hardware, coupled with improved algorithms for acquisition and analysis of the calcium data, have resulted in marked improvements in reproducibility [68, 69]. A study of 1,376 asymptomatic individuals undergoing sequential scanning resulted in an average variability of 15–17% [70]. A recent study using a newer scanning protocol revealed a mean interscan variability of 16–19.9% and a median variability of 4–8% [71]. Another improvement was the introduction of the volume score, relying on isotropic interpolation to better quantitate the CAC burden and further reduce variability [72–74]. 

However, with the more recent MDCT scanners, better image quality significantly contributed to improve reproducibility [75]. Currently, median interscan variability is about 7%, and mean interscan variability is 15% with MDCT, allowing serial followup at no closer than 1-year intervals.

### 7.4. CAC Quantification

CAC scanning can be used to noninvasively track changes of coronary atherosclerosis by periodically quantifying CAC [76] and thereby demonstrating the effects of therapy on this process. Several studies have been conducted to evaluate the progression of CAC over time using one of two methodologies for scoring. 

CACP, as measured on cardiac CT, is defined as a hyperattenuating lesion above a threshold of 130 Hounsfield units (HUs) with an area ≥3 adjacent pixels (at least 1 mm^2^). There are currently 2 CT calcium scoring systems widely used: the original Agatston method [76] and the volumetric score method [37]. The Agatston score method involves multiplication of the calcium area by a number related to CT density. Densities between 130 and 199 receive a score of 1, between 200 and 299 a density factor of 2, between 300 and 399 is a 3, and ≥400 a multiplicative factor of 4. A calcium score is reported for a given coronary artery and for the entire coronary system; however, most research studies have reported data related to the summed or total score for the entire epicardial coronary system. Janowitz et al. [66] demonstrated that the score progression was more marked in patients with obstructive CAD compared with patients who had no clinical manifest disease (27% versus 18%). One study, although relatively small (*n* = 81), demonstrated an increase in the CAC score by 24% each year since baseline [61]. A larger study (*n* = 299) with an average of 2.2-year followup demonstrated that CAC scores increased by a mean of 33% per year, predicting that the CAC score will more than double every 2.5 years on average [60]. Mitchell et al [77] followed 347 patients for 1.4 years, demonstrating an annual average increase in CAC scores of 21% in men and 18% in women. A small study of young patients with end-stage renal disease demonstrated a mean calcium score rise of 59% per year, with a doubling of score by 20 months [78].

*Effect of Treatment on CAC Progression*. The controversies related to CAC progression are that there are inconsistent relationships between medications known to be beneficial and rates of CAC progression. Statins reduce clinical cardiac end points across a spectrum of patient populations [79–82]. It seemed intuitive to early researchers that statin therapy should reduce the progression of CAC. Callister et al. produced the first report of CACS being used to assess the effects of statin therapy [83]. They conducted a retrospective study of 149 patients with no history of CAD. Repeat scanning was carried out 12–15 months later. 105 patients received statin, and a net reduction in CAC score was found in the treated group who had final LDL-cholesterol levels <3.10 mmol/L (−7 ± 23%; *P* < 0.001). Treated patients who had an average LDL-cholesterol of ≥3.10 mmol/L had an increase in CAC score (+25 ± 22%; *P* < 0.001); further increases were noticed in untreated patients who had an average LDL-cholesterol of ≥3.10 mmol/L (+52 ± 36%; *P* < 0.0001).

In another prospective study, 66 patients who were not on treatment and whose LDL-cholesterol was >3.4 mmol/L, had a repeat EBCT, and treatment with Cerivastatin 0.3 mg/day was initiated after a mean interval of 14 months [84]. A third examination was carried out after 12 months of treatment; the median annual relative increase in CAC score was 25% during the untreated period versus 8.8% in the treatment period (*P* < 0.0001). 

 In the observational study by Budoff et al. [60], 123 persons with hypercholesterolemia were followed. The participants reporting use of a statin (*n* = 60) had an annual rate of progression of 15%, compared with 39% annual increase in EBT score for the 62 persons in the nontreatment group (*P* < 0.001). This represented a 61% reduction in the rate of progression obtained with statin therapy. A study of 160 persons with established coronary artery disease has demonstrated that treatment of patients with severe hypercholesterolemia has resulted in a slowing of the atherosclerotic CAC process [85]. Combination treatment with simvastatin, niacin, and Vitamin E resulted in an annual calcium score progression of 20% as compared to a median of 40% progression measured in the untreated group.

 Rath and Niedzwiecki [86] demonstrated the effect of nutritional supplements (a combination of vitamins, minerals, and coenzymes) on progression of CAC in 55 persons. Patients treated with the nutritional supplements demonstrated an annual progression of 15%, while untreated patients progressed at a rate of 44%. 

However, prospective randomized trials did not support the earlier observational results that statins could slow CAC progression. St. Francis Heart Study is a double-blinded, placebo-controlled, randomized clinical trial where 1005 asymptomatic men and women aged 50–70 years with CAC score ≥80th percentile for age and gender were randomized to receive either Atorvastatin 20 mg daily and vitamin E (*α*-Tocopherol) 1000 units daily or placebo [87] with mean duration of treatment of 4.3 years. Treatment reduced total cholesterol by 26.5–30.4%, LDL-cholesterol by 39–43%, and triglycerides by 11.2–17%, but had no effect on progression of CAC score. More importantly, the treatment with statins did reduce CV events by 42%  (*P* < 0.05) in those persons taking statins as compared to the placebo group, demonstrating definitively that persons with CAC benefit by treatment with statins. 

In another multicenter, randomized double-blind trial [88], 471 patients had ≥2 cardiovascular risk factors and a CAC score of ≥30 with no history of CAD or evidence of significant coronary stenoses. Patients were randomized to receive either low- (10 mg) or high-dose (80 mg) Atorvastatin over 1 year. LDL-cholesterol was reduced in the 80 mg/day group from 2.7 to 2.2 mmol/L, whereas in the 10 mg/day group, it remained at 2.8 mmol/L. The mean progression in 366 patients corrected for baseline score was 27% in the 80 mg/day group compared with 25% in the 10 mg/day. Thus, no difference was found in the progression between the two groups, and no relationship was found between on-treatment LDL-cholesterol and progression. Accordingly, several factors might explain this conflicting data: (1) first statins may cause microcalcifications, which could increase the CAC score while decreasing the lipid core, (2) longer followup period are needed, or (3) statins might be improving the cardiovascular outcome through other pleiotropic effects rather than mere lowering of the LDL-cholesterol or decreasing the CAC scores.

Several prospective randomized trials of garlic therapy on CAC have shown consistent improvement in CAC progression [89–92], suggesting that the microcalcifications may be the cause of inconsistent data related to statins and progression of CAC. CAC progression adds significant incremental prediction ability of all-cause mortality, after adjustment for time between scans, demographics, risk factors, and baseline CAC scores.It appears that persons with scores >30 can be assessed for progression of CAC, and this adds incremental information regarding future prognostic risk. Though utilization of repeat CT testing to estimate an individual's risk associated with CAC “change” appears to be of value, a better understanding of what therapies may be of benefit and how clinicians should utilize these data in clinical practice remains to be determined. 

## 8. Radiation

The radiation exposure associated with CAC scans has been significantly reduced, now comparable to 1-2 mammograms (~1 mSv) [15, 93–97] while providing important information about the subject coronary risk and cardiac anatomy. Measuring CAC progression requires sequential CT scans, with a cumulative radiation exposure. Prior reports have raised concern about the excess risk of cancer with such an approach [98]. However, such predictions are outdated as current gating technology reduces the radiation dose substantially, with an expected dose of <1 millisievert (mSv) per scan [99]. Further advances have reduced the CAC dose to as low as 0.6 mSv, lower than screening mammography [99, 100]. 

## 9. Conclusions

The cumulative data provides strong confirmatory evidence that CAC is a strong predictor of events and that CAC progression is associated with future cardiovascular events and that, as radiation doses are being reduced to a minimum, may be a useful tool in the prevention armamentarium to assess atherosclerosis progression noninvasively. Based on available published evidence, CAC has been incorporated into the ACC/AHA guidelines for screening of asymptomatic individuals for CVD. CAC has been advocated as a screening tool for risk assessment of asymptomatic adults at intermediate risk (10%–20% 10-year risk), low-to-intermediate risk (6%–10% 10-year risk), and patients with diabetes [2]. 

A scientific statement from the AHA [15] agree that with the statement from the AHA perspective paper [57], which stated that “…with a prior probability of a coronary event in the intermediate range (≥6% in 10 years but ≤20% in 10 years), a calcium score of >100 would yield a post-test probability in virtually all such patients greater than 2% per year, that is, a level similar to that in secondary prevention, or a ‘coronary risk equivalent.'” Therefore, all patients with CAC >100 should be considered for statin therapy, aspirin, and possibly ACE inhibition, given the increased cardiovascular risk associated with this level of coronary atherosclerosis, concurring with the current NCEP Adult Treatment Panel (ATP) III guidelines [30]. This supports the conclusions of the Prevention Conference V and the AHA report [15] that high coronary calcium scores confirm increased risk for future cardiac events: “measurement of coronary calcium is an option for advanced risk assessment in appropriately selected persons. In persons with multiple risk factors, high coronary calcium scores (e.g., >75th percentile for age and sex) denote advanced coronary atherosclerosis and provide a rationale for intensified LDLC lowering therapy. Moreover, measurement of coronary calcium is promising for older persons in whom the traditional risk factors lose some of their predictive power.” The severity of CAC can identify asymptomatic patients at high risk for CAD. The resulting calcium score in the respective category can be used to stratify an asymptomatic person's risk for a future cardiac event. Accordingly, either modification of risk factors or further testing can be pursued to reduce the likelihood of such an event from happening [58]. For persons at high risk, an accurate and noninvasive measure of atherosclerosis could allow better assessment of processes associated with disease progression and of therapies to prevent the progression of atherosclerosis and clinical CAD [59].
